# Supplementary material for: LiMMCov: An interactive research tool for efficiently selecting covariance structures in linear mixed models using insights from time series analysis
Source: PLoS One. 2025 Jun 11;20(6):e0325834. doi: 10.1371/journal.pone.0325834 (PMC12157095; doi:10.1371/journal.pone.0325834)
Supplement: S1 File — Yule-Walker equations for AR(1) and AR(2) processes. (DOCX) [file pone.0325834.s001.docx]

**S1 File. Appendix A.** Yule-Walker Equations for AR(1) and AR(2) Processes

The Yule-Walker equations provide a systematic method for estimating the parameters of autoregressive (AR) processes. This appendix demonstrates the application of Yule-Walker equations for AR(1) and AR(2) processes, presenting the derivation of the equations, parameter estimation, and the resulting correlation matrices [1,2].

## **Yule-Walker Equations**

For an AR($p$) process, the Yule-Walker equations are given by:

$$\gamma_{k}=\sum_{i=1}^{p} \phi_{i}\gamma_{k-i}+\sigma^{2}\delta_{k0}, k=0,1,2,\ldots,p$$

where:

- $\gamma_{k}$: Autocovariance at lag $k$
- $\phi_{i}$: AR coefficients
- $\sigma^{2}$: Variance of the white noise error term
- $\delta_{k0}$: Kronecker delta, which is 1 when $k=0$ and 0 otherwise

The autocorrelation function (ACF) $\rho_{k}$ is defined as the autocovariance normalized by the variance:

$$\rho_{k}=\frac{\gamma_{k}}{\gamma_{0}}$$

Rewriting the Yule-Walker equations in terms of the autocorrelation function, we have:

$$\rho_{k}=\sum_{i=1}^{p} \phi_{i}\rho_{k-i}, k=1,2,\ldots,p$$

## **AR(1) Process**

For an AR(1) process ($p=1$), the Yule-Walker equation simplifies to:

$$\gamma_{1}=\phi_{1}\gamma_{0}$$

Dividing through by $\gamma_{0}$, we obtain:

$$\rho_{1}=\phi_{1}$$

The correlation matrix for an AR(1) process with $n$ observations is:

$$\mathbf{C}_{AR\left( 1 \right)}=\left[ \begin{matrix} 1 & \phi_{1} & \phi_{1}^{2} & \cdots& \phi_{1}^{n-1} \\ \phi_{1} & 1 & \phi_{1} & \cdots& \phi_{1}^{n-2} \\ \phi_{1}^{2} & \phi_{1} & 1 & \cdots& \phi_{1}^{n-3} \\ \vdots& \vdots& \vdots& \ddots& \vdots\\ \phi_{1}^{n-1} & \phi_{1}^{n-2} & \phi_{1}^{n-3} & \cdots& 1 \end{matrix} \right]$$

## **AR(2) Process**

For an AR(2) process ($p=2$), the Yule-Walker equations are:

$$\gamma_{1}=\phi_{1}\gamma_{0}+\phi_{2}\gamma_{1}$$

$$\gamma_{2}=\phi_{1}\gamma_{1}+\phi_{2}\gamma_{0}$$

Dividing through by $\gamma_{0}$, we obtain:

$$\rho_{1}=\frac{\phi_{1}}{1-\phi_{2}}, \rho_{2}=\frac{\phi_{1}\rho_{1}+\phi_{2}}{1-\phi_{2}}$$

The correlation matrix for an AR(2) process with $n$ observations is:

$$\mathbf{C}_{AR\left( 2 \right)}=\left[ \begin{matrix} 1 & \rho_{1} & \rho_{2} & \cdots& \rho_{n-1} \\ \rho_{1} & 1 & \rho_{1} & \cdots& \rho_{n-2} \\ \rho_{2} & \rho_{1} & 1 & \cdots& \rho_{n-3} \\ \vdots& \vdots& \vdots& \ddots& \vdots\\ \rho_{n-1} & \rho_{n-2} & \rho_{n-3} & \cdots& 1 \end{matrix} \right]$$

**References**

1. Shumway RH, Stoffer DS. Time Series: A Data Analysis Approach Using R. Time Series: A Data Analysis Approach Using R. 2019. 1–259 p.

2. Chatfield C, Xing H. The Analysis of Time Series: An Introduction with R, Seventh Edition. The Analysis of Time Series: An Introduction with R, Seventh Edition. 2019. 1–398 p.
