# Supplementary material for: LiMMCov: An interactive research tool for efficiently selecting covariance structures in linear mixed models using insights from time series analysis
Source: PLoS One. 2025 Jun 11;20(6):e0325834. doi: 10.1371/journal.pone.0325834 (PMC12157095; doi:10.1371/journal.pone.0325834)
Supplement: S2 File — R Code for longitudinal data simulation and estimation of AR parameters. (DOCX) [file pone.0325834.s002.docx]

# **S2 File. Appendix B:** R Code for Simulation of Longitudinal Data and Estimation of AR Parameters

This appendix provides the R code used to simulate longitudinal data under different correlation structures, including Compound Symmetry (CS), Autoregressive Order 1 (AR1), and Autoregressive Order 2 (AR2) matrices. Additionally, functions for estimating AR parameters using the Yule-Walker equations and constructing the AR(2) correlation matrix are provided.

## **Function to Estimate AR Parameters Using Yule-Walker Equations**

# The function requires time series data and the number of AR parameters to be estimated

ar_parameters <- function(tsdata, p){

tsacf <- acf(tsdata, plot=F)$acf

r = c(tsacf[1:p,1,1])

R = dae::mat.banded(r, p, p)

rho = c(tsacf[2:(p+1),1,1])

rho = matrix(rho, p, 1)

# AR parameter estimates

phi.hat = (solve(R)) %*% rho

# White noise variance estimate

tsacf2 <- acf(tsdata, plot=F, type="covariance")$acf

sigma.sq.hat = tsacf2[1,1,1] * (1 - t(rho) %*% solve(R) %*% rho)

return(list(phi.hat, sigma.sq.hat))

}

# Example use case for time series data y

phi.hat <- as.vector(unlist(ar_parameters(y, 2)[1]))

## **Function to Construct AR(2) Correlation Matrix**

# Function to estimate the autocorrelation matrix using the AR(2) parameters

ar2_corr_matrix <- function (phi.hat, dimen) {

if (length(phi.hat) < 2)

stop("Must supply two values in phi.hat")

if (phi.hat[1] + phi.hat[2] >= 1)

stop("AR(2) series is not causal")

if (phi.hat[2] - phi.hat[1] >= 1)

stop("AR(2) series is not causal")

if (abs(phi.hat[2]) >= 1)

stop("AR(2) series is not causal, parameter phi[2] must be between -1 and 1")

corrs <- vector(mode = "numeric", length = dimen)

corrs[1] <- 1

corrs[2] <- phi.hat[1] / (1 - phi.hat[2])

corrs[3] <- (phi.hat[1]^2 - phi.hat[2]^2 + phi.hat[2]) / (1 - phi.hat[2])

for (k in 4:dimen) {

corrs[k] <- phi.hat[1] * corrs[k - 1] + phi.hat[2] * corrs[k - 2]

}

ar2_corr <- dae::mat.banded(corrs, dimen, dimen)

return(ar2_corr)

}

## **Simulation of Longitudinal CD4 Count Data**

The simulations generate repeated measures data for 100-150 subjects over 10 time points, incorporating various fixed and random effects.

*Common Simulation Parameters*

Total subjects (nsubj): 100-150

Time points per subject (nt): 10

Fixed effects:

Baseline CD4 count (alpha0): 300

Time effect (beta_time): Positive slope (5-15)

Gender effect (beta_gender): Negative (-10)

Age effect (beta_age): Positive (2)

Weight effect (beta_weight): Positive (1.5)

Marital status (beta_marital) and WHO stage (beta_WHO): Categorical effects

### *Simulation 1: AR(1) Correlation Structure*

# Set simulation parameters

set.seed(202310)

nsubj <- 150 # Number of subjects

nt <- 10 # Number of time points

phi <- 0.8 # AR(1) autocorrelation parameter

# Subject-level covariates

age <- rnorm(nsubj, mean = 32.7, sd = sqrt(9.6))

gender <- rbinom(nsubj, 1, prob = 0.6)

weight <- rnorm(nsubj, mean = 50, sd = sqrt(8.6))

marital_status <- sample(1:4, size = nsubj, replace = TRUE,

prob = c(0.1, 0.3, 0.4, 0.2))

WHO_stage <- sample(1:4, size = nsubj, replace = TRUE,

prob = c(0.2, 0.15, 0.55, 0.1))

# Random intercepts and AR(1) residuals

random_intercepts <- rnorm(nsubj, mean = 0, sd = 10)

e_ij <- generate_ar1_residuals(nsubj, nt, phi, sigma_epsilon = 25)

# Simulate CD4 count

df$CD4_count <- alpha0 +

df$random_intercept +

beta_time * df$time +

beta_gender * df$gender +

beta_age * df$age +

beta_weight * df$weight +

df$e_ij

### *Simulation 2: Compound Symmetry (CS) Correlation Structure*

# Set simulation parameters

set.seed(202310)

nsubj <- 100 # Number of subjects

nt <- 10 # Number of time points

rho <- 0.5 # Compound symmetry correlation

# Create compound symmetry covariance matrix

cs_cov_matrix <- create_compound_symmetry_matrix(nt, sigma_epsilon, rho)

# Generate correlated residuals

residuals <- matrix(0, nrow = nsubj, ncol = nt)

for (i in 1:nsubj) {

residuals[i, ] <- mvtnorm::rmvnorm(1,

mean = rep(0, nt),

sigma = cs_cov_matrix)

}

# Simulate CD4 count

df$CD4_count <- alpha0 +

df$random_intercept +

beta_time * df$time +

beta_gender * df$gender +

beta_age * df$age +

beta_weight * df$weight +

beta_marital[df$marital_status] +

beta_WHO[df$WHO_stage] +

df$residual

### *Simulation 3: AR(2) Correlation Structure*

# Set simulation parameters

set.seed(202310)

nsubj <- 100 # Number of subjects

nt <- 10 # Number of time points

# AR(2) parameters

phi.hat <- c(-0.9726550, -0.4476774)

sigma_epsilon <- 30 # Residual error standard deviation

# Create AR(2) correlation matrix

ar2_cor <- ar2_corr_matrix(phi.hat, nt)

ar2_cov <- sigma_epsilon^2 * ar2_cor

# Simulate residuals using Cholesky decomposition

e_ij <- matrix(0, nrow = nsubj, ncol = nt)

for (i in 1:nsubj) {

epsilon_uncorr <- rnorm(nt, mean = 0, sd = 1)

e_ij[i, ] <- chol(ar2_cov) %*% epsilon_uncorr

}

# Simulate CD4 count

df$CD4_count <- alpha0 +

df$random_intercept +

beta_time * df$time +

beta_gender * df$gender +

beta_age * df$age +

beta_weight * df$weight +

df$e_ij

#### Data Storage

Each simulation saves two files:

- - CSV file: `simdata_[structure].csv`
  - RData file: `simdata_[structure].RData`

# Select relevant columns

simdata_[structure] <- df[, c("id", "time", "age", "gender", "weight", "marital_status", "WHO_stage", "CD4_count")]

write.csv(simdata_[structure], file = "simdata_[structure].csv")

save(simdata_[structure], file = "simdata_[structure].RData")
