# Supplementary material for: LiMMCov: An interactive research tool for efficiently selecting covariance structures in linear mixed models using insights from time series analysis
Source: PLoS One. 2025 Jun 11;20(6):e0325834. doi: 10.1371/journal.pone.0325834 (PMC12157095; doi:10.1371/journal.pone.0325834)
Supplement: Attachment — Submitted filename: Response to Reviewers.docx [file pone.0325834.s004.docx]

Mohamed R. Abonazel, Academic Editor

PLOS ONE

March 28, 2025

Dear Editor and Reviewer(s),

Thank you for taking the time to review our work (PONE-D-23-37290) and providing us with insightful feedback on ways to strengthen our paper. We have incorporated the suggested changes and addressed each point below.

We believe that the revised version better communicates the novelty and significance of our work. We are confident that our web application stands as a valuable addition to the existing body of knowledge in this field.

We hope that the revised version meets your expectations.

Thank you for your time and consideration.

On behalf of all the authors,

Yours sincerely,

Perseverence Savieri

Corresponding Author

Biostatistics and Medical Informatics Research Group (BISI)

Vrije Universiteit Brussel (VUB), Brussels 1090, Belgium

[Perseverence.Savieri@vub.ac.be](mailto:Perseverence.Savieri@vub.ac.be)

**Manuscript ID:** PONE-D-23-37290

**Manuscript title:** LiMMCov: An Interactive Research Tool for Efficiently Selecting Covariance Structures in Linear Mixed Models Using Insights from Time Series Analysis

**Editorial comment**

**We note that Figures 3,4,5,6 and 7 in your submission contain copyrighted images. All PLOS content is published under the Creative Commons Attribution License (CC BY 4.0), which means that the manuscript, images, and Supporting Information files will be freely available online, and any third party is permitted to access, download, copy, distribute, and use these materials in any way, even commercially, with proper attribution. For more information, see our copyright guidelines: http://journals.plos.org/plosone/s/licenses-and-copyright.**

We want to explain that these figures are not copyrighted third-party images; rather, they are screenshots generated from the LiMMCov application, which we developed ourselves. Hence, they belong to us, and we consent to publishing them under the CC BY 4.0 license. We have updated all the figure legends to clarify that these images are original screenshots from our research tool. To meet PLOS requirements, we confirm that all figures were checked using the Preflight Analysis and Conversion Engine (PACE) digital diagnostic tool as recommended.

**Reviewer #1:**

*Major areas of improvement*

**1- Real‐world longitudinal studies often have missing time points or irregular sampling intervals. While the manuscript mentions that LiMMCov “requires complete data,” it would benefit from guidance or references on handling missingness before importing.**

Thank you for this suggestion. We first want to point out that multiple imputation is only one of various strategies for handling missing data, and each approach (e.g., full information maximum likelihood, inverse probability weighting) has specific requirements and assumptions, particularly regarding sample size or missing not-at-random (MNAR) data. We have revised the Discussion section of the manuscript (lines 526-532) to provide references that discuss different missing-data methods in more detail, helping readers choose techniques best suited to their study’s characteristics. In addition, we emphasize that LiMMCov focuses on the selection of covariance structures, so data preprocessing and the choice of imputation or missing-data handling approach lie outside the app’s scope. Nevertheless, we have included notes in the ‘Dataset’ and ‘Manual’ tabs and the app’s manual to remind users to address missingness appropriately (for instance, via multiple imputation or other suitable methods) before uploading data to LiMMCov. This ensures the validity of subsequent analyses and aligns with best practices in longitudinal data handling.

- Molenberghs G, Fitzmaurice G, Kenward MG, Tsiatis A, Verbeke G. Handbook of missing data methodology. CRC Press; 2014.
- Nooraee N, Molenberghs G, Ormel J, Van den Heuvel ER. Strategies for handling missing data in longitudinal studies with questionnaires. J Stat Comput Simul [Internet]. 2018;88(17):3415–36. Available from: <https://doi.org/10.1080/00949655.2018.1520854>
- Ji L, Chow SM, Schermerhorn AC, Jacobson NC, Cummings EM. Handling Missing Data in the Modeling of Intensive Longitudinal Data. Struct Equ Model [Internet]. 2018;25(5):715–36. Available from: <https://doi.org/10.1080/10705511.2017.1417046>
- Heymans MW, Twisk JWR. Handling missing data in clinical research. J Clin Epidemiol [Internet]. 2022;151:185–8. Available from: <https://doi.org/10.1016/j.jclinepi.2022.08.01>

**2- Although the authors note that performance was not “thoroughly tested” for data sets with few repeated measures or small samples, this point warrants stronger emphasis. In practice, many longitudinal studies have 2–3 time points. The utility and stability of time‐series diagnostics (particularly PACF) in such constrained designs might be limited.**

We appreciate the reviewer’s focus on studies with minimal time points. We have revised the manuscript (lines 533-543) to emphasize more strongly that the stability and interpretability of time-series diagnostics (such as the PACF) are indeed limited when the number of repeated measures is very low. In these scenarios, a compound symmetry structure may be the most practical option. Our simulations suggest that LiMMCov performs well with at least three repeated measures per subject and a sample size of 50 participants or more, but we acknowledge the need for further research to confirm performance in smaller designs. We believe these clarifications better highlight the app’s limitations in real-world settings with constrained data.

**3- While combining AIC/BIC with diagnostic plots is valuable, the paper might clarify the limitations of purely empirical selection (e.g., the possibility of local optima or how random effects can alter apparent residual patterns). Specifically, clarifying how random‐effects structures and residual covariance structures interact would strengthen the discussion.**

We appreciate your attention to this important interplay between random effects and residual covariance structures. We have added a paragraph (lines 550-564) in the Discussion section acknowledging that specifying multiple or complex random effects can already capture part of the serial correlation, making the simultaneous specification of a detailed marginal covariance structure more prone to overparameterization or non-convergence. Moreover, we emphasize that our approach offers a practical, data-driven mechanism to arrive at a defendable model, though not necessarily the most complex or “true” model. This recognizes the inherent limitations of empirical selection and underscores that LiMMCov should be used as part of a careful model-building process that balances fit, interpretability, and feasibility in real-world datasets.

**4- The manuscript focuses on common information criteria (AIC, BIC) and the partial autocorrelation function. However, other approaches (e.g., cross‐validation, robust standard errors, or specialised small‐sample corrections like AICc) are only briefly mentioned. It might be worth highlighting or demonstrating how these can be integrated within the app or used in tandem with its outputs.**

We acknowledge the reviewer’s interest in additional model selection and diagnostic approaches. In response, we now include AICc in LiMMCov’s model-fit comparison table, specifically for smaller-sample scenarios where AICc may be more appropriate than AIC or BIC. Regarding cross-validation, we believe it is more aligned with predictive objectives, whereas LiMMCov’s primary goal is to identify and compare different covariance structures in a longitudinal framework. As such, we currently do not implement cross-validation. We acknowledge that robust standard errors could provide further insights when model assumptions are questioned, but this extends beyond the scope of our tool, which emphasizes comparative model diagnostics rather than fully robust estimation. Nonetheless, we have expanded the Discussion section (lines 484-490) to note that users may apply robust standard errors or cross-validation in external workflows, potentially in conjunction with LiMMCov output, if their study objectives require these additional methods.

**5- Many longitudinal data sets may exhibit more complex correlation patterns (e.g., moving‐average components or partially nested random effects). It would be prudent to acknowledge that LiMMCov currently does not handle MA(q) or ARMA(p,q) structures.**

Thank you for highlighting this. We have added a note in the Discussion (lines 540-543) explicitly acknowledging that LiMMCov does not currently support MA(q) or ARMA(p,q) structures. We aimed to focus on the most commonly employed covariance formulations (e.g., AR(p), CS) to keep the interface and model selection process manageable for most typical longitudinal studies. Nonetheless, we acknowledge the need for more advanced correlation structures in future updates.

**6- As the authors note, random effects and residual covariance structures can be complementary. An overly complicated random‐effects structure could obscure the patterns in the residuals. Readers might need guidance on balancing the complexity of each part of the model.**

We appreciate the reviewer’s emphasis on finding the right balance between random effects complexity and the modelling of residual correlations. However, LiMMCov follows the marginal formulation perspective, guiding the marginal covariance structure selection. We acknowledge this limitation by linking it to comment (3). In the revised Discussion (lines 550-564), we clarify that LiMMCov is designed specifically to help users select among candidate residual covariance structures and does not handle the full hierarchical model-building process, which includes random effects. We note that excessive complexity in the random-effects portion can mask meaningful residual correlations..

**7- Provide a quick “decision tree” in the manual: for instance, “If your residual plot shows a gradually decaying correlation with lag, consider AR(1). If it shows oscillation, consider AR(2). If a flat correlation for all lags, consider CS,” etc. This can be an excellent at-a-glance guide.**

We appreciate this suggestion to offer a concise “decision tree” for guiding users. We have incorporated such a guide into the “Manual” tab of the LiMMCov application (printscreen is shown below), where users can find recommendations on when to consider AR(1), AR(2), Compound Symmetry, or other structures based on their residual plots and autocorrelation patterns. We believe this resource enhances the user experience by clarifying how to interpret diagnostic outputs and choose an appropriate correlation structure more confidently.

**8- Enhance reporting of model diagnostics. In addition to the final AIC/BIC, incorporate:**

**o Model Convergence Warnings: Indicate if the model has trouble converging, prompting the user to revise initial guesses.**

**o Random-effects Diagnostics: Although residual correlation is the focus, briefly mentioning random-effects variance components can reassure advanced users about model fit.**

We appreciate the reviewer’s suggestions and are pleased to confirm that LiMMCov already provides checks and warnings when models fail to converge, prompting users to adjust their inputs accordingly. Regarding random-effects diagnostics, we respectfully note that LiMMCov is rooted in the marginal formulation and thus focuses on modelling the residual covariance matrix rather than examining different random-effects structures in detail (as also discussed in our responses to Comments 3 and 6). In the Discussion section, we acknowledge that random-effects variance component diagnostics can be valuable for advanced users, but these features currently lie outside the scope of our tool’s primary objective.

**9- While the authors discuss covariance structures, many practical problems also feature heteroskedastic residuals (where variance changes over time). Adding a note on how LiMMCov might be extended or used when residual variances are not assumed constant (e.g., varIdent in nlme) would expand its scope**.

We appreciate the reviewer’s observation regarding heteroskedastic residuals. Although our approach initially assumes stationarity, we have added an option in LiMMCov to allow for both homoscedastic and heteroscedastic (varIdent()) residual variances for the selected covariance structure. This update acknowledges that real-world longitudinal data often exhibits changing variance over time. However, extending LiMMCov to a broader range of heteroscedastic models offered under variance function classes (i.e. varClasses in nlme) is currently out of scope (lines 543-548).

**10- To identify boundary cases, encourage broader usage and external testing (e.g., including more diverse example data sets or user-submitted open data).**

Thank you for this suggestion. In the current version, we provide multiple example datasets, both simulated (AR1, AR2, CS) and from a published study, to demonstrate typical use cases. We also allow users to upload their own datasets with varying designs and complexities. To further expand testing and identify boundary cases not captured by our initial examples, we welcome user-submitted open data, and we have added a not in the app to encourage those willing to make their datasets publicly available to share them. We plan to maintain an online repository of such user-contributed datasets, enabling broader community engagement and facilitating more extensive real-world testing of LiMMCov.

*Minor issues*

**1- The word “models” is repeated (“LMMs models”), the word models is in LMMs. Please remove models after LMMs.**

Thank you for noticing this; we have removed the word models after LMMs (lines 1-2).

**2- Lines 84–85 (“However, the AIC does not offer insight ... black box identification”): The sentence could be clarified.**

Thank you for pointing out the need for clarification. We have revised the sentence to emphasize that although AIC provides a numerical basis for comparing models, it does not reveal the underlying reasons why one model’s covariance structure may be preferable. This lack of interpretability can be problematic for researchers who wish to understand the justification behind the chosen model. The revised text now reads (lines 87-89): “However, while AIC is useful for comparing competing models, it offers limited insight into why one model outperforms another, effectively making the selection process a ‘black box’ for identifying the model structure.”

**3- While the text defines many terms (e.g., AR(p), CS, etc.), some readers may benefit from succinct definitions or references to standard texts the first time each structure is named. For example, a short descriptive bullet might clarify that “Compound Symmetry (CS) implies a constant correlation ρ between any two observations for the same subject.”**

Thank you for highlighting that more detail or references would be helpful when introducing each covariance structure. We have revised the manuscript by adding a concise definition and relevant references the first time each structure is mentioned (lines 55-60). This should assist readers who are less familiar with AR(1), compound symmetry (CS), and other covariance structures in understanding their basic assumptions and where to find further details.

**4- The results section might be clearer with subheadings like “3.1 Residual Diagnostics,” “3.2 Model Fit Comparison,” “3.3 Final Selected Model,” to provide an at-a-glance roadmap.**

Thank you for suggesting clearer subheadings in the Results section. We have reorganized the presentation to include subheadings such as “Residual Diagnostics,” and “Final Selected Model,” while retaining the logical steps to illustrate how the Shiny app is used from data loading to final report generation. This approach should provide readers with both a step-by-step workflow and an at-a-glance roadmap of the key analytical steps, as recommended.

**5- Certain sentences would read more fluently if combined or slightly rephrased, especially around lines 468–476, where the text shifts from discussing SAS/SPSS limitations to referencing time-series approaches. Ensure each paragraph transitions smoothly from one concept to the next.**

Thank you for your suggestion. We have revised the manuscript, and the sentences around these lines (now lines 500-509) have been rephrased to improve their fluency and transitions.

**6- Overall, the text is quite readable but can be made more concise by removing repeated points and ensuring that each paragraph targets a single theme.**

Thank you. We have revised the manuscript to address repeated points and ensure that each paragraph targets a single theme. Specifically, we consolidated overlapping discussions, removed redundant sentences, and reorganized the text to improve clarity and flow, as can be seen in the track changes. We believe these changes have resulted in a more concise and coherent manuscript.
