# Supplementary material for: LiMMCov: An interactive research tool for efficiently selecting covariance structures in linear mixed models using insights from time series analysis
Source: PLoS One. 2025 Jun 11;20(6):e0325834. doi: 10.1371/journal.pone.0325834 (PMC12157095; doi:10.1371/journal.pone.0325834)
Supplement: Attachment — Submitted filename: Response_to_Reviewers_auresp_2.docx [file pone.0325834.s005.docx]

Mohamed R. Abonazel, Academic Editor

PLOS ONE

May 6, 2025

Dear Editor and Reviewers,

Thank you for taking the time to review our revised submission (PONE-D-25-03886R1) and providing us with feedback on ways to strengthen our paper. We have incorporated the suggested changes and addressed each point below.

We believe that the revised version better communicates the novelty, significance of our work and potential avenues for future work. We are confident that our web application stands as a valuable addition to the existing body of knowledge in this field.

We hope that the revised version meets your expectations.

Thank you for your time and consideration.

On behalf of all the authors,

Yours sincerely,

Perseverence Savieri

Corresponding Author

Biostatistics and Medical Informatics Research Group (BISI)

Vrije Universiteit Brussel (VUB), Brussels 1090, Belgium

[Perseverence.Savieri@vub.ac.be](mailto:Perseverence.Savieri@vub.ac.be)

**Manuscript ID:** PONE-D-25-03886R1

**Manuscript title:** LiMMCov: An Interactive Research Tool for Efficiently Selecting Covariance Structures in Linear Mixed Models Using Insights from Time Series Analysis

**Reviewer #2:**

*Minor areas of improvement*

**1. The Yule-Walker equations have some limitations that the authors should consider:
The accuracy of the estimates depends on the assumption that the time series is stationary, meaning that its statistical properties do not change over time. If the time series is non-stationary, differencing or other transformations may be required before applying the Yule-Walker method.
The Yule-Walker estimates are based on the sample autocorrelation function, which can introduce sampling error, especially for small sample sizes.**

Thank you for pointing this out. From a linear mixed-model viewpoint, the residual covariance structures that LiMMCov compares (i.e, AR(1), AR(p), compound symmetry, etc.) from the nlme package (Pinheiro et al., 2023), all embed the assumption of weak stationarity. Because this assumption underlies the likelihood for any LMM fitted with such structures, it was natural to maintain it when generating synthetic data for our validation study. Therefore, we first generated stationary AR(p) processes (verifying that all characteristic roots lay outside the unit circle) and then applied the Yule-Walker equations to obtain the corresponding AR coefficients. These coefficients defined the correlation matrices used to simulate longitudinal datasets for benchmarking LiMMCov. We have clarified in the Methods section (lines 223-225) that the Yule-Walker procedure was applied under weak-stationarity assumptions. We also acknowledge, now in the Discussion (lines 553-561), that Yule-Walker estimators rely on the sample autocorrelation function and can be imprecise in very small samples. However, this potential sampling error pertains only to the data-generation phase of our simulations; LiMMCov itself remains agnostic to how the covariance matrix is obtained and can be applied to empirical data regardless of the method used to estimate or specify residual correlations. Hence, the core functionality of the app is not altered.

**2. The PACF function in Figure 6 has several values that exceed the confidence interval limits at lags 9 and 16. The component's behavior decreases harmonically, which suggests — based on the inversion of AR models into MA models — the possibility of the existence of an MA component rather than higher-order AR components.**

We appreciate the reviewer’s time-series perspective. The out-of-band spikes at lags 9 and 16 indeed suggest that a moving-average (MA) component, or an ARMA(p,q) process, could underlie the residual pattern. LiMMCov, however, is intentionally limited to autoregressive and other commonly used stationary covariance structures in linear mixed models; modelling explicit MA or ARMA components is beyond this first release's scope. As noted in the Discussion (and echoed by Reviewer 1), the absence of MA/ARMA covariance structures is a current limitation (lines 541-544) and an important direction for future development as we continue integrating time-series methods into LMM frameworks. In the meantime, users who suspect MA behaviour can either analyse the series externally with dedicated time-series software or approximate the effect by increasing the AR order within LiMMCov, while recognising the approximation’s limitations.

**3. The authors correctly note that there are still "grey areas" to be explored, but I would recommend also discussing the possibility of a stochastic trend.**

We appreciate this insightful recommendation. LiMMCov is intended as an entry point for the linear-mixed-model (LMM) community to adopt time-series concepts; accordingly, the current version assumes that, once an appropriate mean structure is specified, the residuals are weakly stationary. Any stochastic (unit-root) trend, therefore, needs to be removed or modelled in the fixed-effects part of the LMM (e.g., polynomial time terms) before LiMMCov’s covariance-structure selection is applied. Because an ARIMA(p,d,q) process with d>0 becomes an ARMA(p,q) process after differencing, such non-stationary behaviour cannot be accommodated simply by specifying an “ARIMA covariance matrix.” Supporting integrated components would instead require additional modelling layers (e.g., automatic differencing or state-space extensions), and evaluating their usefulness within the LMM framework would first necessitate a feasibility or sensitivity study, work we regard as future research rather than part of the present release. We have revised the Discussion section (lines 541-544 and 563–573) with parts that (i) explain how users can handle a stochastic trend with the existing tool and (ii) identify the incorporation of extended ARMA-type covariance structures as a logical, but still prospective, enhancement to LiMMCov.

**4. The violation of stationarity in the series variance — which is closely linked to the covariance matrix — should not be overlooked. This leads to the use of ARCH models, which opens a new “window of opportunity” for future articles. This aspect should also be mentioned among future research directions!**

We agree that conditional heteroskedasticity, often observed as “volatility clustering,” where large residuals tend to be followed by large residuals, constitutes an important avenue for future work. At present, LiMMCov can accommodate unconditional heteroskedasticity via varIdent(), but it does not estimate models in which the residual variance evolves as an explicit stochastic process driven by past squared errors (e.g., ARCH/GARCH behaviour). We have therefore added a sentence in the Discussion (lines 547-551) that (i) acknowledges volatility clustering as a motivation for ARCH models and (ii) lists ARCH/GARCH covariance structures as a promising avenue for future development and methodological research.
